# Iliopsoas hematoma presenting with sudden knee extensor weakness: A case report

**DOI:** 10.1097/MD.0000000000023497

**Published:** 2020-12-11

**Authors:** Jae Hoon Kim, Seung Don Yoo, Dong Hwan Kim, Young Rok Han, Seung Ah Lee

**Affiliations:** a Department of Physical Medicine and Rehabilitation, Kyung Hee University Hospital at Gangdong; b Department of Physical Medicine and Rehabilitation, College of Medicine, Kyung Hee University, Seoul, Republic of Korea.

**Keywords:** femoral neuropathies, hematoma, psoas muscles

## Abstract

**Rationale:**

Hematoma of the iliopsoas muscle is a rare condition. Prolonged pressure conditions due to hematoma of the femoral nerve can cause severe pain in the affected groin, hip, and thigh, and quadriceps weakness. We report a rare case of a spontaneous iliopsoas muscle hematoma that caused sudden femoral neuropathy.

**Patient concerns:**

A 71-year-old woman presented sudden left hip pain and knee extensor weakness. The pain was aggravated with left hip extension. She had a bilateral total hip replacement surgery due to avascular necrosis. She was diagnosed as mild stenosis of the cerebral artery and took aspirin to prevent cerebral artery atherosclerosis.

**Diagnosis:**

A hip computed tomography scan demonstrated a suspicious fluid collection at the left iliopsoas bursa. We considered the possibility of lower limb weakness due to neuralgic amyotrophy and performed electromyography and enhanced lumbosacral magnetic resonance imaging (MRI). Electromyography finding showed left femoral neuropathy of moderate severity around the inguinal area was diagnosed. On MRI, left iliopsoas bursitis or hematoma, and displacement of the left femoral nerve due to the iliopsoas bursitis/hematoma were observed.

**Intervention:**

Ultrasonography (US)-guided aspiration of the left iliopsoas hematoma was performed. We started steroid pulse therapy for 8 days.

**Outcomes:**

After US-guided aspiration and steroid pulse therapy, the patient's knee extension motor grade improved from grade 1 to 2, and the pain was slightly reduced. At 3 weeks after the aspiration procedure, her hip flexion motor grade had improved from grade 3+ to 4 at follow-up.

**Lessons:**

Imaging studies are fundamental to diagnose of iliopsoas hematoma. Electromyography examination plays an important role in determining the prognosis of patients and lesion site. Despite the negligible change in sitting position, hematoma can develop. Physicians should consider hematoma that cause femoral neuropathy.

## 1 Introduction

Hematoma of the iliopsoas muscle is a rare condition. It occurs when patients receive anticoagulation therapy or in patients with either inherited or acquired coagulation disorders^[1]^ or a direct injury, tumor, or other growth blocking or trapping the nerve. These prolonged pressure conditions due to hematoma on the nerve can cause severe pain in the affected groin, hip, and thigh, and quadriceps weakness. Femoral nerve palsy due to iliopsoas hematoma is a rare condition.^[2]^ The iliopsoas muscle consists of 2 muscles with different areas of origin. Iliopsoas muscle is innervated by the femoral nerve (iliacus) and direct branches of the lumbar plexus (psoas). The iliopsoas muscle is an important walking muscle and leads to spine turning/rotation.

We report a rare case of spontaneous iliopsoas muscle hematoma that caused sudden femoral neuropathy. We conducted a literature review of previous case reports of quadriceps weakness in patients without any direct traumatic history and coagulation abnormality.

Written informed consent was obtained from the patient for publication of this case report and accompanying images.

## 2 Case report

A 71-year-old woman presented with sudden left hip pain and knee extensor weakness. While sitting on a chair, slightly leaning to right side, she felt an electrical shock-like pain (from the left inguinal area to the anteromedial thigh and knee) while shifting weight to the left side. The pain was aggravated upon left hip extension. She had a bilateral total hip replacement surgery due to avascular necrosis. She was diagnosed as mild stenosis of the cerebral artery and took aspirin to prevent cerebral artery atherosclerosis. On physical examination, the manual motor grade (manual muscle test [MMT]) was 3+ for left hip flexion (Medical Research Council of Great Britain) and 1 for left knee extension. The patellar tendon reflex decreased at the left knee. Tenderness was observed at the mid-portion of the left hip groin area. Considering her history, she had a neuropathic pain and no direct trauma that could cause fractures or tear.

**Figure 1:**
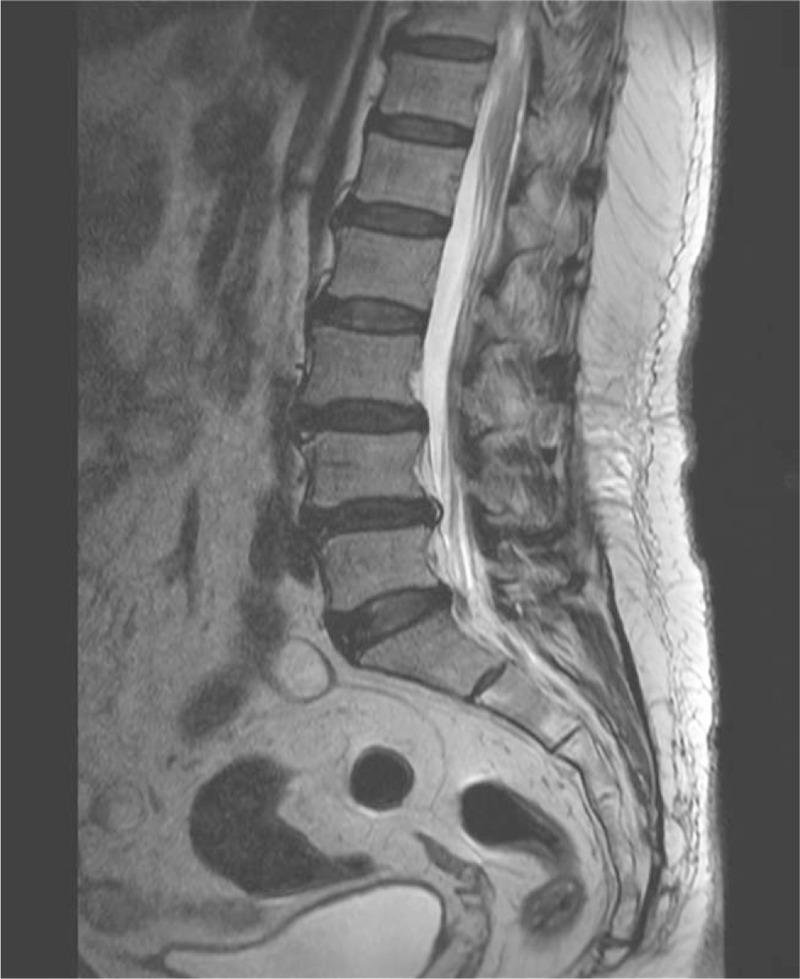
Lumbosacral T2-weighted magnetic resonance image (sagittal view) of L3–L4 and L4–L5 degenerative spondylolisthesis, disk degeneration, and posterior annular fissure at L4–L5.

**Figure 2:**
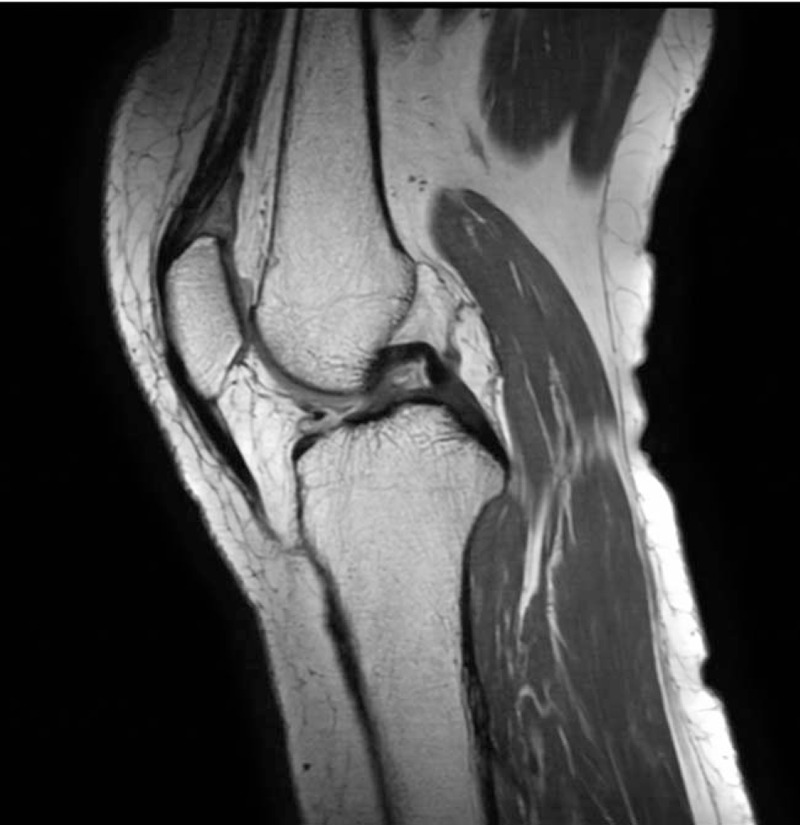
Left knee magnetic resonance image of the edematous change of the suprapatellar fat pad with increased signal intensity in T2-weighted image and posterior bulging contour of the fat pad (suggesting suprapatellar fat pad edema syndrome), and an irregular low signal line with inner high signal at the subchondral portion of the lateral femoral condyle (posterior aspects).

**Figure 3:**
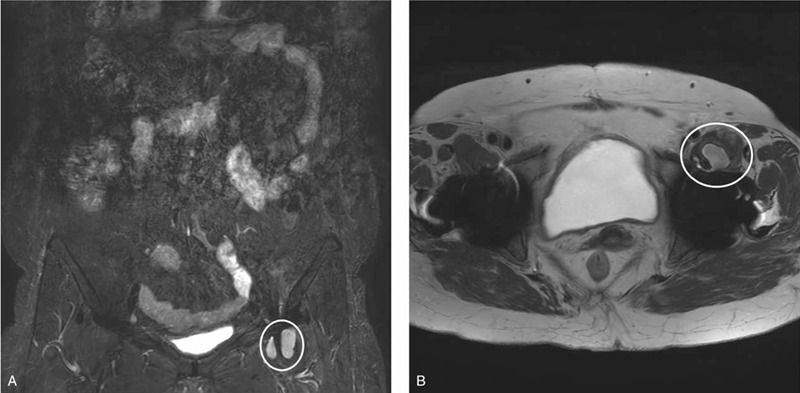
Enhanced lumbosacral plexus magnetic resonance imaging (MRI) scan of the left iliopsoas bursitis or hematoma (1.9 cm in thickness). The displacement of left femoral nerve due to iliopsoas bursitis/hematoma (white circle). (A) Coronal view of the T2-weighted MRI scans of the lumbosacral plexus. (B) Sagittal view of T2-weighted MRI scans of the lumbosacral plexus.

First, magnetic resonance imaging (MRI) was performed to rule out radiculopathy-induced weakness. On lumbar spine MRI, L3–L4, and L4–L5 degenerative spondylolisthesis (Fig. 1) and no definite causes of weakness were found. Second, the knee extensor motor grade was 1, which was much worse than the hip flexor motor grade of 3. To rule out rupture of the left patellar tendon, left knee MRI was performed (Fig. 2). However, no definite abnormality of the left patellar tendon was found. Only an edematous change of the suprapatellar fat pad was found. Blood test results showed no definite abnormality (prothrombin time [PT], 11.2 seconds; PT%, 97%; PT international normalized ratio, 1.02; activated partial thromboplastin time, 28.4 seconds). Until then, no clear causes of weakness of the left hip and knee were observed on the spine and knee imaging, and no evidence with clotting disorders was shown. Third, hip computed tomography (CT) scans were performed to rule out hip injury itself, although the patient had a history of low-velocity, low-amplitude position change (Fig. 3). On the hip CT scan, no fracture was found, but a fluid collection at the left iliopsoas bursa (approximately 3.8 × 3.2 cm in size), suspicious of iliopsoas bursitis, which is a common finding after total hip replacement arthroplasty, was observed. Lastly, the physicians considered the possibility of lower limb weakness due to neuralgic amyotrophy (lumbosacral plexitis, based on a sudden onset of extreme pain followed by motor weakness) and performed electromyography and enhanced lumbosacral MRI to confirm the neuropathic condition precisely. Sensory nerve conduction studies were unobtainable for the left saphenous nerve. Other sensory studies had normal results. The motor nerve conduction studies showed a small amplitude at the left common peroneal nerve (recording at the extensor digitorum brevis, 62.5% compared with the right side) and femoral nerve (recording at the vastus medialis, 13.75% compared with the right side). A needle electromyography showed a discrete recruitment pattern at the left vatus medialis and iliopsoas, no activity at the left rectus femoris, and a reduced recruitment pattern at the left extensor digitorium brevis, adductor halluces, peroneal longus, medial gastrocnemius, gluteus medius, and maximus. The findings from the electromyographic studies were compatible with left femoral neuropathy alone, with severe axonotmesis around the inguinal level, rather than lumbosacral plexopathy. The enhanced lumbosacral MRI scan indicated left iliopsoas bursitis or hematoma, which displaced the left femoral nerve (Fig. 3).

**Figure 4:**
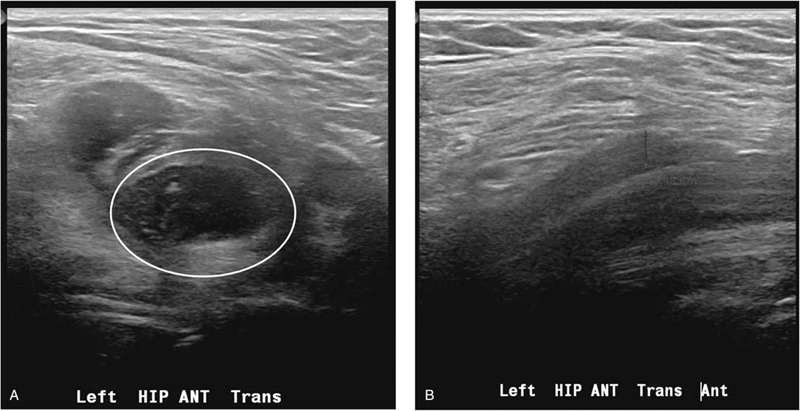
Ultrasonography-guided aspiration of the left iliopsoas hematoma. Approximately 10 cc of dark bloody fluid was aspirated (red circle, revealed hematoma in the iliopsoas muscle). (A) The white circle indicates the hematoma at the iliopsoas muscle on ultrasonography. (B) After removal of the hematoma, the thickness of the left iliopsoas hematoma decreased 0.3 cm.

Finally, considering the electromyography and lumbosacral MRI findings, the fluid collection near the iliopsoas muscle level was the most likely cause of the left knee extensor weakness. Hip ultrasonography was performed for aspiration, which decreased the burden of the mass effect on the left femoral nerve. Ultrasonography-guided aspiration was performed. The bursitis or hematoma at the lower portion of the left iliopsoas muscle was approximately 6 × 2 × 1.4 cm (Fig. 4A). Approximately 10 cc of dark bloody fluid was aspirated, and the thickness of the hematoma decreased from 1.4 to 0.3 cm (Fig. 4B). Immediately after the aspiration, the knee extensor motor grade improved from grade 1 to grade 2, and the hip flexor motor grade was stationary (grade 3+). Pain was slightly reduced. We started steroid pulse therapy for 8 days, starting prednisolone administration at a dose of 80 mg and then tapering it to 10 mg. An additional blood test was recommended to evaluate for hematological disorders because the patient had only minor physical stress at the onset of symptoms. Peripheral blood smear and measurements for fibrin degradation product, D-dimer, lupus anticoagulant, thrombin time, complement 3, complement 4, anti-thrombin III, and factor XIII (functional) were performed. As a result, no abnormality was found in the coagulation factor test. One week after the aspiration, a follow-up nerve conduction study was performed. The result was slightly improved in terms of the left femoral nerve latency (recording at the vastus medialis) of the compound motor action potential. At 3 weeks after the hematoma aspiration, the motor grade improved from 3+ to 4 for hip flexion and from 2 to 2+ for knee extension.

## 3 Discussion

We report a case of femoral neuropathy caused by a hematoma of the iliopsoas muscle, which decreased the knee extension motor grade and increased the pain at the groin area. The incidence of spontaneous iliopsoas hematoma is 0.1% in the general population and 0.6% in elderly patients receiving anticoagulation therapies or affected by coagulopathies.^[20]^ Hematoma of the iliopsoas muscle has been reported to occur after trauma or hip arthroplasty, and even in normal patients; it has been reported primarily in patients with hemophilia and those receiving heparin or oral anticoagulation treatment.^[1,3–5,7]^ The causes of femoral neuropathy include medical conditions such as diabetes mellitus, gout, osteoarthritis, pelvic tumors, pelvic and upper femoral fractures, labor, surgical procedures in the abdomen or pelvis, aneurysms of the femoral artery, femoral nerve compression due to hematoma after rupture of an abdominal aortic aneurysm, and uremia.^[11]^

Direct trauma to the pelvis or hip joint hyperextension can cause muscle tears, which lead to iliopsoas hematoma. When the clinical onset is sudden or the symptoms gradually worsen, the iliopsoas hematoma could compress femoral nerve.^[12]^ The typical clinical symptoms include groin pain that can migrate to the lumbar region or thigh, hip flexion and quadriceps paralysis, the absence or reduction of the patellar tendon reflex, and absent or reduced sensation of the anterior thigh region.^[13]^

Femoral neuropathy caused by iliopsoas hematoma can be suspected in the appropriate clinical setting and presentation. Diagnosis is usually confirmed radiographically. On plain radiography of the abdomen and pelvis, an enlarged psoas shadow may be observed. Ultrasonography has been used with some success but can be limited by body habitus and underlying bowel gas. MRI is sensitive for the detection of retroperitoneal processes and is useful to rule out nerve root compression.^[14,15]^ MRI has also high sensitivity for the detection of small hematomas but is not widely available. Computed tomography is the most common imaging tool.^[2,16,17]^

Previous studies reported that the clinical symptoms of femoral neuropathy depend on its level and severity. A complete lesion above the inguinal ligament results in knee extension paralysis, severe hip flexion weakness, and sensory loss over the anteromedial thigh and medial leg. The patellar tendon reflex is lost. Lower or partial lesions result in milder and more-restricted syndromes such as isolated sensory phenomena or quadriceps motor weakness only. In our case, we confirmed lower or partial lesions of the femoral nerve compression throughout the imaging study and electromyography.^[18]^

The treatment of iliopsoas muscle hematoma with associated femoral neuropathy remains controversial because of its rarity. The treatment depends on the onset time, and volume and degree of neurological impairment. A conservative approach is justified for hemodynamically stable patients with smaller hematomas, with moderate neurological symptoms, and without active bleeding.^[5,6,10]^ However, in the case of active bleeding, transarterial embolization can be a treatment option.^[7–9]^ In a previous study, because hematoma formation due to active bleeding has lower morbidity, transarterial embolization should be considered instead of surgery. For large hematomas with severe motor function inhibition, if the lesion progresses or neurological worsening is evident, surgical treatment by decompression and drainage should be considered.^[1,10]^ In addition, another study^[1]^ suggested that when surgical hematoma evacuation was performed at the first identification of neuropathy, a full return of function is more likely to occur, and patients treated conservatively for an extended period despite the presence of femoral neuropathy continue to have prolonged neurological deficit despite eventual intervention. In one case series, all the patients who had a femoral motor deficit and were conservatively treated or underwent surgical drainage after 48 hours had a permanent femoral nerve distribution paresis.^[10]^ More recently, another study recommended early fasciotomy of the iliopsoas muscle. The authors reasoned that early fasciotomy with or without hematoma evacuation should be considered to provide rapid decompression and to minimize the chance of permanent nerve injury.^[7]^

In another previous study, young age and low axonal loss were favorable prognostic factors. The study also showed a distinction between predominant axon loss and dysfunction due to conduction block by estimating the degree of axonal loss, determining the time course of recovery, and suggesting blocked nerve fibers (neurapraxia) as the predominant type of injury. Patients with axonal loss of >50% took a long time to recover (poor outcome), suggesting severe axonotmesis as the predominant type of injury. Mixed types of injury had low level of axonal loss and showed an excellent but delayed recovery.^[19]^

For diagnosis of iliopsoas hematoma, early recognition with a high level of suspicion is required. The treatment decision about this clinical entity depends on the speed of onset, size of the hematoma, and degree of neurological impairment.

In this case, the patient sat in a chair and leaned slightly to the right and moved to the left. This was a negligible change for the development of a hematoma. It spent a considerable amount of time to find the cause of the weakness. In retrospect, she complained of neuropathic pain in the area of the anterior cutaneous branch of the femoral nerve, and the motor power of hip flexion was preserved as compared with that of knee flexion relatively. This means that the hematoma at the lower level of the iliopsoas muscle (the branch to the iliacus of the femoral nerve, which was relatively saved) caused the marked weakness of knee extension. Immediately after the hematoma aspiration, the knee extensor power improved but recovered incompletely. This treatment was performed 16 days after the onset of symptoms.

## 4 Conclusion

The symptoms of femoral neuropathy resemble those of lumbar radiculopathy and hip pathology. In our case, for patients with pain such as an electrical shock-like pain at the inguinal area after position change, imaging studies (e.g., lumbar and hip imaging and ultrasonography) are fundamental to differentiate diagnosis to avoid misdiagnosis. Electromyographic examination plays an important role in diagnosing the injury site and prognosis. In the case of lower limb weakness and pain caused by position change without a special medical history, an accurate diagnosis should be made by appropriate imaging and electromyography.

## Author contributions

**Conceptualization:** Jae Hoon Kim, Seung Don Yoo, Dong Hwan Kim, Young Rok Han, Seung Ah Lee.

**Data curation:** Jae Hoon Kim, Dong Hwan Kim, Seung Ah Lee.

**Formal analysis:** Jae Hoon Kim.

**Methodology:** Young Rok Han, Seung Ah Lee.

**Project administration:** Seung Don Yoo.

**Resources:** Dong Hwan Kim, Young Rok Han.

**Supervision:** Seung Don Yoo, Young Rok Han, Seung Ah Lee.

**Validation:** Seung Ah Lee.

**Writing – original draft:** Jae Hoon Kim, Seung Don Yoo, Dong Hwan Kim, Young Rok Han, Seung Ah Lee.

**Writing – review & editing:** Jae Hoon Kim, Seung Don Yoo, Dong Hwan Kim, Young Rok Han, Seung Ah Lee.
